# Implementation of START (STrAtegies for RelaTives) for dementia carers in the third sector: Widening access to evidence-based interventions

**DOI:** 10.1371/journal.pone.0250410

**Published:** 2021-06-02

**Authors:** Sarah Amador, Penny Rapaport, Iain Lang, Andrew Sommerlad, Naaheed Mukadam, Aisling Stringer, Nicola Hart, Shirley Nurock, Gill Livingston

**Affiliations:** 1 University College London, London, United Kingdom; 2 University of Exeter, Exeter, United Kingdom; 3 Alzheimer’s Society, London, United Kingdom; Cardiff University, UNITED KINGDOM

## Abstract

Family members remain the main care providers for the increasing numbers of people with dementia, and often become depressed or anxious. In an implementation research project, we aimed to widen access to Strategies for RelaTives (START), a clinically and cost-effective intervention for the mental health of family carers, by laying the foundations for its implementation in the third sector. We used the integrated Promoting Action on Research Implementation in Health Services (i-PARIHS) framework to guide implementation of START, a manual-based, individually-delivered, multicomponent eight-session coping strategy intervention. We interviewed a maximum variation sample of twenty-seven stakeholders from the English Alzheimer’s Society (AS), about possible difficulties in management, training, and delivery of START. We trained and supervised three AS dementia support workers in different locations, to each deliver START to three family carers. Two researchers independently coded pre-intervention interviews for themes. We assessed intervention feasibility through monitoring delivery fidelity, rating audio-recordings from 1–5 (5 being high) and interviewing facilitators, family carers and AS managers about their experiences. We assessed effectiveness on family carers’ mental health using the Hospital Anxiety and Depression Scale (HADS) before and after receiving START (scores 0–42). We changed START’s format by reflecting carer diversity more and increasing carer stories prominence, but core content or delivery processes were unchanged. All carers received START and attended every session. The mean fidelity score was 4.2. Mean HADS-total score reduced from baseline 18.4 (standard deviation 7.4) to follow-up 15.8 (9.7). Six (67%) carers scored as clinically depressed on baseline HADS and 2 (22%) at follow-up. Facilitators and carers rated START positively. Appropriately experienced third sector workers can be trained and supervised to deliver START and it remains effective. This has the potential for widened access at scale.

## Introduction

Around 40% of family carers of people living with dementia have clinical depression or anxiety [1, 2]. Maintaining the mental health of family carers of people with dementia is important for them, and may help them to continue caring at home [3]. We developed START (Strategies for RelaTives) to improve family carers’ mental health and quality of life, by using helpful coping strategies and reducing dysfunctional strategies [4]. We demonstrated clinical and cost-effectiveness immediately and up to six years later in a randomised controlled trial (RCT) [5–8] START is an eight-session, manual-based coping strategy intervention. Therapists are trained graduate psychologists, supervised by a clinical psychologist. Carers are seen one-to-one and supported to fill in their own manual, thus individually tailoring the intervention. The sessions comprise: behavioural management, communication strategies, identifying and changing unhelpful thoughts, accessing emotional support, increasing pleasant events, relaxation, future planning, practice between sessions and developing a maintenance plan [9]. Carers keep their manual, stress reduction audio-recordings and continue to use what works for them [10].

We designed START to be scalable; it is manual-based for consistent delivery, and delivered by non-clinically qualified staff. However, high demand from carers coupled with lack of resources currently fuel waiting lists for START in health services. Also, some family carers prefer non-healthcare settings as they do not see themselves as ill.

In the United Kingdom, so-called “third sector” organisations are neither public nor private organisations (and sometimes called “not-for-profit” organisations) contracted by the state through the commissioning process to supply public services, which are delivered by paid staff. Third sector organisations are high profile with large reach to family carers, so those that are hard to reach in healthcare settings, may benefit from START’s availability in the third sector. We consequently worked with the Alzheimer’s Society (AS)—the UK’s largest dementia charity—who identified Dementia Support Workers (DSWs), non-clinically trained support staff who work with carers and people with dementia in local communities, to learn to deliver START.

### Aims and objectives

We aimed to widen access to START by laying the foundations for implementation in the third sector using the AS as an exemplar. Our objectives were to adapt and deliver START for delivery within existing AS structures, evaluate its feasibility and effectiveness, and inform implementation of evidence-based dementia interventions in these settings.

## Methods

London—West London & Gene Therapy Advisory Committee Research Ethics Committee Reference 18/LO/0369 gave approval. All participants gave written, informed consent.

### Guiding framework and outcomes

We used the integrated Promoting Action on Research Implementation in Health Services (i-PARIHS) framework [11, 12] to guide implementation. We considered five components:

1. Characteristics of the intervention believed to influence implementation (positively and negatively).
2. Characteristics of the organisation’s inner context (e.g. readiness for implementation) and outer context (e.g. external policy) that enable or constrain implementation.
3. Individuals and teams involved–intervention recipients, and staff training and support needs and how they may support or resist innovation.
4. Facilitation–changes required to clarify the manual for the third sector and to align with its priorities and practice, and additional activities required to deliver the intervention in a different context.
5. Outcomes–staff and carers’ opinion in qualitative interviews; and quantitative measurement of carer mood, quality of life and services used to examine if outcomes remain similar to the effectiveness trial.

### Qualitative interviews pre-intervention

We developed a semi-structured interview guide (see S1 Appendix) using the Consolidated Framework for Implementation Research (CFIR) [13, 14] which provides a pragmatic structure for systematically assessing the first three components listed above. We interviewed a range of stakeholders aiming for a maximum variation sample, and transcribed in full the digital recordings of the interviews. We then used this information for facilitation (component 4) to enable successful implementation.

### Recruitment, training and supervision of DSWs

We collaborated with AS senior leadership to ensure alignment with existing service delivery. Prior to delivery, we engaged the senior leadership team and organised a dissemination question and answer event about START at their headquarters. We provided area managers with a one-page evidence sheet about START for commissioners [15]. The AS research translation team asked area managers in South-East England for expressions of interest from DSWs in delivering START. We detailed the time needed for training and supervision and the financial reimbursement for the DSWs’ time. We asked that those expressing interest obtain manager approval and confirm their capacity to spend time training and seeing carers for START. We recruited, trained and supervised three DSWs as facilitators, ensuring we addressed their and the organisations’ needs identified in the pre-intervention interviews. DSWs committed to identifying three family carers of people with dementia who were able to attend eight sessions, delivering START to them, attending supervision and audio-recording a randomly allocated session for fidelity. They also agreed to take part in a post-delivery interview with the research team.

DSWs received four three-hour group training sessions over two days with the lead clinical psychologist (PR) in how to deliver the intervention, clinical skills and potential clinical dilemmas and effective use of supervision. DSWs also reported spending around ten hours in self-directed learning between sessions reading, learning and practicing the manual. Members of the clinical team (GL, NM, AS, PR) spent a further four three-hour sessions with the DSWs over two days. The DSWs delivered each DREAMS session by role-play, one DSW being the carer and one delivering the session. Clinicians completed a structured checklist as to whether core competencies had been reached and that individual elements of the sessions were delivered. They also discussed common dilemmas which occurred and good practice. They then signed off DSWs as competent to deliver the intervention. Throughout delivery, DSWs received group clinical supervision fortnightly by phone with a clinical psychologist.

### Family carers’ quantitative assessment

We collected demographic information from all participants including age, sex, ethnicity, relationship to the patient (e.g. spouse or child), level of education, work and living situation.

To allow comparison with the original randomised controlled trial [6], we completed the following measures at baseline and post-intervention:

- The Hospital Anxiety and Depression Scale (HADS) [16, 17]. The HADS-total score (HADS-T; possible scores ranging from 0 to 42, higher scores indicating more symptoms) was the primary outcome in the original RCT and it aligns with diagnostic criteria for depression [6]. The scale also generates the HADS-depression (HADS-D) and HADS-anxiety (HADS-A; scores from 0 to 21). The anxiety and depression scales dichotomise as ‘case’ and ‘non-case’, with a cut-off point of 8/9.
- The Health Status Questionnaire (HSQ) [18, 19] mental health domain, measuring health-related quality of life (QoL). Scores range from 0 to 100, with higher scores indicating better QoL.
- The Client Service Receipt Inventory (CSRI) [20], which comprehensively covers health and social services use.

### Intervention fidelity

Staff audio-recorded one START session per carer selected through randomisation by the study manager. Though facilitators may have their own style of delivery, manuals include specific points to make verbatim, points of discussion and prompts to be included in each session. Two researchers not involved in START delivery, rated sessions independently for fidelity using a standard session-specific checklist of around 18 items devised for the original trial by PR and GL [6]. An overall fidelity score for each session was then given by the rater considering whether or not the therapist was ‘keeping the carer focused on the manual’. Possible scores ranged from 1, meaning ‘not at all’, to 5, meaning ‘very focused’.

### Qualitative interviews post-intervention

We interviewed carers and facilitators post-intervention about the process of delivery [21], and their experience of receiving and delivering START. We digitally recorded and transcribed interviews in full.

### Analysis

We used the i-PARIHS structure to analyse interviews and describe changes needed to implement START, considering organisational policy, staff training and support needs, manual changes and intervention delivery. We open-coded initial interviews for themes, then developed a coding framework [22]. Two researchers double coded each interview using NVivo 12 to organise data and discussed discrepancies to reach agreement. Any disagreements were discussed with the wider team to be resolved. We reported the scores and changes on the quantitative assessments before and after START delivery. To assess acceptability, post-intervention interviews with carers and staff were analysed using a previously developed model to consider views about START [6] and the Theoretical Framework of Acceptability [23] following steps outlined above.

## Results

### Pre-intervention interviews

We interviewed 27 Alzheimer’s Society stakeholders across inner-city, suburban and rural locations in the South-East of England including senior operations managers, line managers and frontline staff (see Table 1 for roles descriptions).

**Table 1:** Pre-intervention interviews–stakeholder titles and roles.

Title	Number	Roles
Operations Manager	4	Provides leadership for the wider area encompassing multiple boroughs, leads and coordinates organisational activity at a local level
Commissioner	1	Part of Clinical Commissioning Groups (CCG) set up to organise the delivery of National Health Services in England, including community health services
Services Manager	5	Local team manager, oversees management and delivery of services including services budgets, meeting contractual requirements, and maintaining external relationships,
Dementia Support Manager	2	Provides day to day direct support to a team of Dementia Support Workers
Day Support Manager	1	Management of day support services including the management of staff and volunteers, and all other operational aspects of the Day Support Service.
Dementia Support Worker/Dementia Adviser	12	Dedicated member of staff giving one to one support, information and guidance to people with dementia or their carers and wider family or friends. Dementia Support Workers are predominantly community-based (e.g. person with dementia’s own home) and have a smaller caseload than Dementia Advisers. Dementia Advisers are usually the first point of contact for the person with dementia and their carer, and can refer to a Dementia Support Worker if more support is needed
Group Facilitator	2	Deliver Alzheimer’s Society groups services, including peer support, Dementia Cafes, and Singing for the Brain

They discussed characteristics of the intervention, people involved in delivery and the third sector context which they thought would influence implementation. Where applicable, we discuss modifications (e.g. to the manual) and facilitation activities in response to support delivery of START. Themes are presented in detail in the following section.

#### Characteristics of the intervention

Interviewees thought the strength and quality of the evidence underpinning START would facilitate third sector implementation, as local health and social services commissioners prioritise evidence-based, cost-effective programmes.

*You’ve proved it works so this is a big step*. *It’s not a* ‥ *pilot* ‥ *that would be a very different matter*. *(Operations Manager 1)*

*The programme is evidence-based and it can really enhance people’s lives*, *or improve their quality of life*, *I think it’s not really that difficult … we can show that it’s good value for … the scarce resources … we have (Commissioner 1)*

Perceived value for money, linked to the intervention being limited to eight sessions, giving carers the skills to cope with caring long-term, was appealing. This contrasted with, for example, peer support groups that carers continue to attend long-term.

*… Once you’re in the group service you could be in the same group for two or three years so* ‥ *you’re not reaching so many (Dementia Service Manager 1)*

*You know*, *if we’ve given them this time initially*, *I think it would save us time later*. *If we’re skilling people up to manage their own situation*,‥ *that’s got to be a good thing (Operations Manager 1)*

*(…) with dementia if [a good] early intervention is not there*, *it will cost more (…) they will run into crisis so they will end up in hospitals*, *care homes*, *which always cost more (Services Manager 3)*

Staff, however, were concerned that START may increase the demand for support.

*Potentially a one-hour session could end up with an extra five support needs … How do you handle …*. *continuing demand* …? *Obviously*, *the point of the programme is to develop the self-coping strategies*, *but… (Dementia Support Manager 1)*

They also thought that carers may find it difficult to be away from the person they cared for but had concerns about how to manage interactions between the carer-person with dementia dyad, if present during the sessions. Addressing these concerns was already integral to START training and supervision and we ensured this was explicit [15, 24].

#### Characteristics of the organisational context

Stakeholders reported wide variation in local service structure and described how this may influence future uptake and delivery.

*(…) it’s different in every area (…) You know*, *I would embrace it but that might not be the case in other areas of the country*. *(Operations Manager 1)*

Frontline and managerial staff expressed concern over their organisation’s readiness to implement START in terms of available resources beyond the study, leadership engagement and access to knowledge and information about START, and thought that implementation of START on a national scale would require endorsement at all management levels as part of the Society’s core offer.

*[I]n central office they have all these grand schemes and you’re going*, *well*, *how would that work in operation*? *(Services Manager 5)*

*We don’t have enough time to embed the change before another change comes (…) it’s important to be*‥ *frontrunners within the field of dementia; however*, ‥ *there’s only so much that we can* ‥ *change at once (…) people need that period of stabilization*. *(Operations Manager 4)*

*“I would want the time to prepare (…) There is an expectation (…) that you absorb [innovations] into* … *your normal everyday cases and [START] is running alongside it (…) I’d need to know how that would work (Dementia Support Worker 3)*

*If it increased workloads*, *I would struggle with it (…) provisions are going to be needed… for dementia support worker during [delivery] (Operations Manager 1)*

Finally, staff expressed concerns over how START fitted with existing procedures, for example referral processes, safeguarding procedures and electronic record keeping but also felt that it fit within their service delivery model

*(…) we do case work and they should be working with 20–25 people at any given time*. *And so*, *it fits in with that*. *(Operations Manager 3)*

Regarding the outer organisational context, stakeholders thought START might give their organisation a competitive edge.

*So if this is seen to be*, *you know [the Alzheimer’s Society is] doing this and no one else is*, *that’s a really good thing*. *(Operations Manager 2)*

A potential barrier to START’s implementation in the third sector, in terms of external policy and incentives, was the charity’s dependence on local health and social care commissioning to fund services.

*Our only difficulty (…) it’s quite a significant difficulty is our dependence on these local authority contracts…at a local level they’re our real clients*. *(Operations Manager 2)*

#### Facilitation

We responded to perceived contextual barriers by engaging the senior leadership team at the outset and throughout and making materials available to present to commissioners [15]. We also troubleshooted practical difficulties in recruiting DSWs and delivery, addressing concerns about capacity, training and leadership. In parallel to training and supervision undertaken by the research team, the AS research translation team set up discussions at a senior leadership level about long-term implementation. They also supported internal communication about START through the organisation’s publications.

#### Individuals and teams involved

START was perceived as an intervention that could build on existing strengths in the workforce of frontline staff who are often highly experienced.

*I’m surprised you know these are relatively junior staff*‥ *doing a job less than they could be doing*. *(…) they went to uni (Operations Manager 1)*

*Peer support groups and Dementia Cafés*, *and day support groups* ‥ *meet a need*. *But actually*, *lots of people could deliver that*. *Whereas a dementia support worker* ‥ *know their stuff around dementia*. *We want to focus on really* ‥*supporting [them]*. *(Operations Manager 1)*

Stakeholders saw the potential of START to benefit family carers and staff’s job satisfaction and career progression.

*Maybe looking into some of the psychological elements of it*, *like the behaviours*, *the emotions (…) It gives us more of an understanding of [what] we’re supporting people with (…) (Dementia Support Worker 2)*

*But our experience will tell us that people really want something specific to caring for a person with dementia*. *Because their challenges and their needs are very different (Operations Manager 1)*

Additionally, frontline and managerial staff were confident in staff capability with adequate training and supervision to deliver START.

*Being able to practice it* ‥ *where I feel comfortable (…) have somebody there who had an experience of delivering it already to give me feedback (Dementia Support Worker 2)*

#### Need for changes in manuals

Interviewees found START user-friendly and easy to follow, written in “plain English”, but the manual required modifying illustrations of participants and the names used in vignettes to reflect carer diversity.

*Really easy to read*, *work through*, *work out*, *you know what you’re supposed to be doing*, *yes*, *I liked it (Services Manager*, *SM*, *1)*

*We have different people from different cultural backgrounds and when I go out (…) I’ll give them the [leaflet] that* ‥ *might fit” (Dementia Support Worker 1)*

Staff highlighted the importance of personal “carer to carer” testimonials so we increased their prominence in the manual.

*Somebody’s already done this*, *and they said it was good* ‥ *ideally in quite a specific way*…*It changed the way I looked at the situation*, *and*, ‥, *a*, *kind of*, *carer to carer*‥, *communication style*, *rather than a clinical approach (Services Manager 2)*

We also added information about local resources and more blank pages for carers to make notes.

### Changes to supervision

We adapted supervision for the third sector to include discussion of structured information to record in the AS electronic notes, to fit with existing record keeping procedures and map onto existing organisationally defined outcomes, and procedures about safeguarding concerns. Other adaptations for the setting and carers were addressed during supervision, including delivering sessions by phone for one carer, and delivering sessions in a mixture of English and carers’ native language. We also provided support to DSWs’ managers to address any concerns.

### DSWs and carers

Managers supported five DSWs for training but two were unable to attend set training days. The three participating DSWs each nominated three family carers to receive START, who all consented to the study. DSWs had prior contact with five of the nine carers consented to the study, as the first point of contact for the person living with dementia and their carer, who can refer to a Dementia Support Worker for additional support if needed and for one DSW facilitating a carer support group once a week. Table 2 shows the socio-demographic characteristics of carers who received START and the DSWs who delivered it. The carers were demographically diverse, covering both sexes, and a range of ages, ethnicities and relationships to the person with dementia. One DSW was working towards a degree in Psychology and two had a Masters–one in Health and Social Education and the other in Clinical and Community Psychology. Two reported having gained additional qualifications in counselling or therapy.

**Table 2:** Family carer and dementia support worker socio-demographics.

	Characteristic		
**Family carer (n = 9)**	**Age**	**Mean 56.9 (Standard deviation, SD 12.0)**	**Range 33–75**
	**Sex**	**Female**	**7 (78%)**
		**Male**	**2 (22%)**
	**Ethnicity**	**White UK**	**5 (56%)**
		**South Asian**	**3 (33%)**
		**Mixed White and South Asian**	**1 (11%)**
	**Marital**	**Married**	**7 (78%)**
		**Single**	**1 (11%)**
		**Widower**	**1 (11%)**
	**Education**	**School-level education**	**5 (56%)**
		**Further education**	**4 (44%)**
	**Work situation**	**Full time**	**2 (22%)**
		**Part time**	**5 (56%)**
		**Not working**	**2 (22%)**
		**Retired**	**2 (22%)**
	**Living with person with dementia**	**Yes**	**8 (89%)**
		**No**	**1 (11%)**
	**Relationship with the person living with dementia**	**Spouse/Partner**	**5 (56%)**
		**Daughter/Son**	**3 (33%)**
		**Daughter-in-law/Son-in-law**	**1 (11%)**
**Dementia Support workers (n = 3)**	**Age**	**Mean 42 (SD 16.4)**	**Range 28–60**
	**Sex**	**Female**	**3**
	**Ethnicity**	**White UK**	**1**
		**South Asian**	**1**
		**Black African and Caribbean**	**1**
	**Education**	**School-level education**	**1**
		**Further education**	**2**
	**Years in post**	**4 (SD 2.6)**	**Range 1–6**

### Adherence and fidelity

All carers attended all eight START sessions between December 7 2018 and August 8 2019. Eight received all sessions face-to-face; one received six sessions by phone. Sessions took between 6 and 13 weeks to deliver, with a mean of 9 weeks (standard deviation (SD) = 2.12) and took an average of 75 minutes per session to deliver (min 30, max 120). One carer had less than 7 days between some sessions, finishing in 6 weeks. Each DSW audio-recorded one session for fidelity for each of the eight participants seen face-to-face. Mean fidelity score was 4.2 (SD 0.49).

### Quantitative outcomes

Mean HADS-T score reduced from baseline to follow-up (baseline mean 18.4; SD 7.4, range 5–31 to follow-up mean 15.8; SD 9.7, range 6–31). Six (67%) carers scored as clinically depressed on HADS at baseline and two (22%) at follow-up. Six (67%) carers scored as clinically anxious on HADS at baseline, and four (44%) at follow up. The HSQ mental health improved by 5.8 points from baseline to follow-up (baseline mean 45.3, SD 19.3, to follow-up mean 51.1, SD 16.7).

### Acceptability of the intervention

We interviewed nine family carers of people living with dementia between two and 16 weeks after their last session, and three Dementia Support Workers, two AS line managers and one area manager. Results are discussed in detail in the following section.

#### Carers receiving START

Every carer continued to use between one and two START components post-intervention and used different components, including assertive communication techniques, developing behaviour strategies, increasing pleasant events, changing unhelpful thoughts and relaxation. Behaviour, communication and relaxation techniques, and educational components were considered important. Six carers felt that they received the intervention at the right time in their relative’s disease progression; two felt it was late and one it was early. Carers felt that START and their interaction with their therapist had a positive impact upon their attitude to their relative. Specifically, they had accepted the diagnosis and situation more, and found their feelings were validated. Carers valued the personalised and individually delivered approach:

*in a group (…) I* ‥ *find it very hard to assert myself*…*I can find it quite intimidating (…) If there’s men there as well*, *I just find it a very difficult environment (Carer 1)*.

*I can tell her everything*, *I can explain to her what I want and she can listen properly because it’s only me there (Carer 2)*

Carer also highlighted the skill, knowledge and experience of the DSWs:

*she would come up with ideas*, *obviously just from dealing with so many different people in the same situation*. *No-one else can* ‥, *because no-one deals with people in those situations (Carer 1)*

*she obviously knew what she was talking about*, ‥ *here is someone who is affirming what I am doing and she’s telling me I’m doing well*, *(Carer 3)*

*She’s obviously got an in-depth knowledge of dementia*, *and therefore*, *all the things that I bring up*, *she knows the answer to*. *But it’s the way she presents it to you*, *and encourages you (Carer 4)*

Some aspects of START were liked by most but not all carers, for example one carer did not like the relaxation techniques. Three carers wanted more sessions, or sessions spread out over a longer period. Two relatives of someone with young-onset dementia wanted pictures in the manual of younger couples.

*Seeing pictures of old people*, *we’re different*, *maybe this doesn’t apply to us* ‥ *30 years younger than these people (Carer 5)*

Although part of the message of the manual was that carers should use components which were relevant to them and not all would be, one felt that their partner had no changes in behaviour and that behavioural changes discussed did not apply to him.

*As a couple*, *we talk …*. *And it’s respect on both sides (Carer 6)*

#### Staff involved in START delivery

Participating DSW and managers felt very positive about the intervention, despite initial concerns about their ability to deliver alongside their existing workload.

*I was really nervous* … *Will I be able to make that difference*? *Is this intervention really going to work*? *Am I the only one that loves it*?,. *All my three carers*, *they love the manual*, *their booklets*. *(Dementia Support Worker 4)*

*I found it really quite organic*, *actually*. *Because it was just like any other work that I would be doing (Dementia Support Worker 4)*

*it’s been worth it and I think it’s been really valuable*. *It’s one of these things where it’s*, *obviously it’s time-intensive*, *otherwise you don’t get the quality and you don’t get the depth (Services Manager 5)*

*You can’t just slot it in without considering the time*, *eight sessions to take*. *And not just the sessions; the preparation*, *the organization*, *the flexibility*, *the recording*, *the administration*. *(Dementia Support Worker 5)*

Staff felt that they would only be able to continue to deliver START if the service was commissioned to do so.

*When I think about our strategic ambition to reach more people*, *it will have to be those people that most need it*. *[START’s] not something that we can roll out to everybody (Operations Manager 5)*

The DSWs reported that START provided carers with coping strategies and increased their well-being, and that carers took what worked for them from the intervention.

*it was also about empowering the carer* ‥ *because I’m not always going to be there …my carer was really ready … now we’re finishing this week*, *but she’s very relaxed about that*. *She’s not panicking (Dementia Support Worker 4)*

*every single session* ‥ *was very different in all* ‥*that I delivered with*. *Which means it’s tailor-made to what is important to them*. *(Dementia Support Worker 5)*

*the way in which it can empower is really impressive* (*Services Manager 5)*

*All … were passive communicators*. *They didn’t realise that*. *And they realised that passive communication wasn’t working for them and being assertive worked (Dementia Support Worker 4)*

*And the feedback from the carers is that it does work …*. *It’s nice to know that there is a tool that can help carers that are really struggling or that don’t realise that what they are doing is* … *okay (Dementia Support Worker 6)*

DSWs felt confident delivering the intervention and that they would continue to improve and reduce time taken to deliver individual sessions.

*I think you just get better at it*. *And I think I would continue to get better at it, basically (Dementia Support Worker 5)*

*Every week [carers] coming back they’d say*, *yes I tried this*, *this was really good*, *this behaviour record or this communication record*. *It really helped*. *I see the patterns*, *I’m seeing what’s happening* … *it works*. *(Dementia Support Worker 6)*

Line managers felt that it could be time consuming and difficult with existing workloads, especially initially. Finally, managers and staff felt that clinical supervision of the team was essential.

*And I think you wouldn’t want to do it without that level of supervision*. *So it went beyond my capabilities* … *(Services Manager 5)*

## Discussion

In this study, non-clinically trained third sector support staff successfully delivered START, enjoyed enhancing skills, and saw it working for carers. Core components of START were unchanged, though some aspects were adapted for the local setting and population, which is in keeping with the personalised nature of the intervention, originally conceived to be flexibly delivered, and individually tailored. A recent systematic review [25] of implementation in the third sector of evidence-based interventions did not identify any dementia-related interventions to include. It found perceived staff capability, perceived effectiveness of the intervention and flexibility of the intervention to be factors operating as facilitators in these settings, which is in keeping with results presented here. We found the fit between the intervention and the local service delivery model, and the perceived benefits to stakeholders—both family carers and third sector staff—to be additional factors which may also be useful to consider.

The sessions led by DSWs had good fidelity suggesting that START can be delivered consistently. Carers with a range of socio-demographic characteristics showed excellent adherence in terms of attendance (all carers attended every session), suggesting it was acceptable. In the RCT, of the eight sessions offered, five or more were attended by around 75% of carers in the intervention group. It is possible that higher adherence in this study is related to facilitators having had prior contact with carers and thus potentially having selected carers who were more likely to complete all eight sessions. Implementing a proven intervention with unchanged core therapeutic components and high fidelity in a similar group should lead to similar mental health outcomes. We found this for depression, anxiety, and quality of life, which improved at a level consistent with outcomes observed in the RCT, where it was clinically and cost-effective. In the initial intervention study we recruited all carers and therefore some scored very low or even zero on the HADS. Nevertheless there was a significant difference between those receive START and the treatment as usual group. There was more scope for reduction in score in these third sector carers who all had a relatively high HADS score. This may be because DSWs chose carers they perceived most in need. Post-intervention, all carers reported continued use of START techniques. In the original trial only two-thirds did [6], but interviews were in writing two years post intervention, with no additional probes if a question was not answered. The RCT found START was still effective six years after delivery and we hypothesise this to be through continued use of effective techniques [26]. Six carers felt that they received the intervention at the right time in their relative’s disease progression. The degree of dementia people were living with in this study was not assessed; in the RCT, just over 70% of people with dementia in the intervention group had very mild to mild dementia. Both carers in this study who felt that the intervention had come too late, were considering a transition for their relative to residential care suggesting that START may work best for family members caring for relatives before carers feel they cannot manage at home.

The study has limitations as it was a feasibility and acceptability trial and therefore relatively small sample size. Results though in line with those observed in the RCT were as expected not statistically significant. While the design of the implementation evaluation was conceptualised by an academic outside the core team (IL), post-intervention interviews were conducted by individuals who worked with the research team but did not deliver START. The AS funded the project through a competitive grant which facilitated collaboration. This paper reports on delivery of START in three differing AS localities, to inform spread and scale-up of the intervention across the organisation. Results show the intervention is feasible in the third sector and sets out a model, but early adopters can be more enthusiastic, and effects should continue to be monitored in a wider context. The incidence of depression and anxiety in this group was relatively high (67%) thus increasing the likelihood of a change in score in comparison to previous studies.

## Conclusions

START, an evidence-based intervention, can successfully be delivered in the third sector. Overall, our results suggest that effective implementation of evidence-based interventions in the third sector depends on proper identification and meaningful engagement with relevant stakeholders, collaborative working with local research and development teams for effective knowledge mobilisation within the organisation, consideration as to how the intervention fits within existing practices, and collaboration with intervention recipients to address practical challenges. Future research in line with recent recommendations for implementing evidence-based interventions (EBI) among third sector organisations [25], requires clear guidelines on how to adapt and modify EBIs to different populations so that they can be modified without compromising effectiveness and follow up for fidelity and effectiveness. Wider implementation of START in the third sector should continue to research longer term outcomes and costs.

## Supporting information

S1 Appendix (DOCX)

S1 Checklist (DOC)

S1 File (PDF)

S1 Fig (DOC)
