# Supplementary material for: Implementation of START (STrAtegies for RelaTives) for dementia carers in the third sector: Widening access to evidence-based interventions
Source: PLoS One. 2021 Jun 2;16(6):e0250410. doi: 10.1371/journal.pone.0250410 (PMC8171938; doi:10.1371/journal.pone.0250410)
Supplement: S1 Appendix — (DOCX) [file pone.0250410.s001.docx]

S1 Appendix

**S1 Table –Pre-intervention topic guide**

| **CFIR Construct (definition)** | **Item for frontline staff (i.e. Dementia Support Workers, Dementia Advisers, Group Facilitators)** | **Item for managerial staff (i.e. Services Manager, Dementia Support Manager, Operations Manager)** |
| --- | --- | --- |
| **Intervention Characteristics** | | |
| **Design Quality & Packaging** (Perceived excellence in how the intervention is bundled, presented, and assembled) | What do you think of the START manual (i.e. the way it looked) | |
| **Relative Advantage** (Stakeholders’ perception of the advantage of implementing the intervention versus an alternative solution) | What are current provisions for carers like in the Alzheimer’s Society? How do you think START compares to the Society’s current programmes available to family carers? | |
|  |  | Would you see START replacing or complimenting a current program or process? |
| **Complexity** (Perceived difficulty of the intervention, reflected by duration, scope, radicalness, disruptiveness, centrality, and intricacy and number of steps required to implement) | What do you think of the START intervention/manual (i.e. number and length of sessions)? Is there anything you would change? | |
|  | Do you think there any practical issues that could make it difficult for family carers to participate in START? What could we do to make it easier? |  |
| **Inner Setting** | | |
| **Implementation Climate** (The absorptive capacity for change, shared receptivity of involved individuals to an intervention, and the extent to which use of that intervention will be rewarded, supported, and expected within their organization) | Have you had experience of new approaches being introduced at the Society? How did it go? Any issues? What do you think helped? If there have not been any changes, do you think the Society would be open to new practices such as START? | Thinking about the way things are done at the Alzheimer’s Society’s, do you think it will be easy or hard to introduce a new intervention like START? As a manager, have you found any ways to help introduce new ideas or working practices? What information do we need to give to staff to get them on board with the idea of START? |
| **Relative Priority** (Individuals’ shared perception of the importance of the implementation within the organisation) |  | Are there other changes happening in the Alzheimer’s Society at the moment and, if so, do you think that would affect the implementation of START? |
| ***Compatibility*** (The degree of tangible fit between meaning and values attached to the intervention by involved individuals, how those align with individuals’ own norms, values, and perceived risks and needs, and how the intervention fits with existing workflows and systems) | Could you tell us about how you usually work with carers? Who initiates contact? Do they come to you or do you go to them? Where? How often? Set times or variable? | How well do you think START fits with the Alzheimer’s Society’s work with carers? How would START be integrated into current processes? |
| ***Organizational Incentives & Rewards*** *(*Extrinsic incentives such as goal-sharing, awards, performance reviews, promotions, and raises in salary, and less tangible incentives such as increased stature or respect) | Do you think you would get recognition or appreciation for doing START within your organisation, and if so, how? |  |
|  | What do you think about there being rewards within your organisation for delivering START, e.g. a certificate of competence, a title “START qualified support worker”, and/or inclusion in performance reviews? | |
| ***Leadership engagement*** (Commitment, involvement and accountability of leaders and managers with the implementation) | Who within your organisation would you go for help and support about START if needed? What could your managers and colleagues do to help you and make this work better? | As a manager, what do you think you could be doing to support them? |
| ***Available Resources*** (The level of resources organizational dedicated for implementation and on-going operations including physical space and time) | Where would be the best place for you to deliver sessions? What practical help would you need with delivering START? | We will provide training – what specific training might your support workers need in advance? Once they have started delivering START, what do you think they would need support with? |
| **Characteristics of Individuals** | | |
| **Knowledge & Beliefs about the Innovation (**Individuals’ attitudes toward and value placed on the intervention, as well as familiarity with facts, truths, and principles related to the intervention) | What do you think of the START intervention/manual (i.e. the things it covered)? How well do you think START meets the needs of family carers who come through the Society? What do you think about the proposed supervision by a psychologist? Do you understand what is meant by supervision (Clarify if needed as to what supervision is)? | |
| **Self-efficacy** (Individual belief in their own capabilities to execute courses of action to achieve implementation goals) | Have you ever done anything like START or another psychological intervention before? Would you feel comfortable delivering something like START? What would make you feel more confident?  What training do you think you would need to deliver the START intervention? (refer back to manual and tasks that carers are required to do at home, such as planning for the future)  Is there anything in particular about START that worries you, having talked about it today? |  |
| **Other personal attributes** (A broad construct to include other personal traits) |  | How easy do you think that it will be to get dementia support workers to work in a psychological way? |
| **Process** | | |
| ***Engaging Champions*** (Individuals who dedicate themselves to supporting, marketing, driving through an implementation) |  | Would it help to have implementation leaders or champions? What could they most helpfully do to support START? Who might be good to have in this role? |
| ***Engaging External change agents*** *(*Individuals who are affiliated with an outside entity who formally influence or facilitate intervention decisions in a desirable direction) |  | Are there any other individuals within the Alzheimer’s Society or other organisations we should speak to about introducing START at the Alzheimer’s Society? |
| ***Engaging Key Stakeholders*** *(*Individuals from within the organization that are directly impacted by the intervention, e.g., staff responsible for making referrals to a new program or using a new work process) | Is there anyone else you think we should be speaking to in order to deliver START? | |
| ***Engaging Innovation Participants*** (Individuals served by the organization that participate in the innovation, e.g., patients in a prevention program in a hospital) | What do you think would be a good way to raise awareness among dementia carers about a new practice such as START? Is there anything else that our team could do to help with that, e.g. a particular leaflet | |
|  |  | How will dementia support workers be made aware about START? |
