# Supplementary material for: Implementation of START (STrAtegies for RelaTives) for dementia carers in the third sector: Widening access to evidence-based interventions
Source: PLoS One. 2021 Jun 2;16(6):e0250410. doi: 10.1371/journal.pone.0250410 (PMC8171938; doi:10.1371/journal.pone.0250410)
Supplement: S1 File — (PDF) [file pone.0250410.s003.pdf]

FULL PROTOCOL TITLE OF THE STUDY

Foundation laying to widen access to START (Strategies for RelaTives)

SHORT STUDY TITLE

START Foundation Laying

Chief Investigator:

Gill Livingston, MBChB, FrCPsyhc, MD Professor of Psychiatry of older people, Division of psychiatry, UCL

Supported by:

Alzheimer's society AS-IGF-16-001

Sponsored by:

University College London (UCL)

Protocol version number and date:

Version 1 20.12.2017

R&D / Sponsor Reference Number(s): 17/0829

IRAS Registration Number: 234808

PROTOCOL VERSIONS

Version Stage	Versions No	Version Date	Protocol updated & finalised by;	Appendix No detail the reason(s) for the protocol update
Current	1	20.12.2017	Sarah Amador, Study Manager	
Previous				

DECLARATIONS

The undersigned confirm that the following protocol has been agreed and accepted and that the investigator agrees to conduct the study in compliance with the approved protocol and will adhere to the Research Governance Framework 2005 (as amended thereafter), the Trust Data & Information policy, Sponsor and other relevant SOPs and applicable Trust policies and legal frameworks.

I (investigator) agree to ensure that the confidential information contained in this document will not be used for any other purposes other than the evaluation or conduct of the clinical investigation without the prior written consent of the Sponsor.

I (investigator) also confirm that an honest accurate and transparent account of the study will be given; and that any deviations from the study as planned in this protocol will be explained and reported accordingly.

Chief Investigator:

Signature

Date..07../...02/2018

Print Name(in full): GILL LIVINGSTON

Position: PROFESSOR OF PSYCHIATRY OF OLDER PEOPLE

On behalf of the Study Sponsor:

Signature: Tabitha Kavoi Date: 09/02/2018

Print Name(in full): Tabitha Kavoi

Position: Research Governance Manager

STUDY SUMMARY

**Identifiers**	
IRAS Number	234808
REC Reference No	
Sponsor Reference No	17/0829
Other research reference number(s) (if applicable)	Z6364106/2018/01/57
Full (Scientific) title	Foundation laying to widen access to START (Strategies for RelaTives)
Health condition(s) or problem(s) studied	Family carers of people living with Dementia
Study Type i.e. Cohort etc	Implementation Study
Target sample size	Up to N=65 participants
**STUDY TIMELINES**	
Study Duration/length	36 months
Expected Start Date	February 2018
End of Study definition and anticipated date	October 2020
Key Study milestones	JRO Submission (December 2017) Ethics Submission (February 2018) Recruitment (February 2018) Manuals ready for delivery (October 2018) Delivery of intervention (October 2018-April 2020) Evaluation (June 2019-July 2020) End (October 2020)
**FUNDING & Other**	
Funding	**Alzheimer's Society** c/o Malayka Rahman-Amin Research Translation Manager Alzheimer's Society 43-44 Crutched Friars London EC3N 2AE T: 020 7423 5133
Other support	Recruitment Support from:<br>**Alzheimer's Society** c/o Malayka Rahman-Amin Research Translation Manager Alzheimer's Society 43-44 Crutched Friars London EC3N 2AE T: 020 7423 5133<br>**London Dementia Clinical Network** c/o Dr Dan Harwood, Clinical Director 115 Denmark Hill Maudsley Hospital | Denmark Hill | London | SE5 8AZ T: 020 3228 1595<br>**Camden and Islington NHS Foundation Trust** Memory Service c/o Dr Vincent Kirchner, Medical Director, email Vincent.Kirchner@candi.nhs.uk<br>**Local community organisations** in Bradford to support recruitment of family carers c/o Co-applicant Dr Kathryn Lord Research Fellow at the Centre for Applied Dementia Studies Email: k.lord1@bradford.ac.uk T:01274 232306
**STORAGE of SAMPLES (if applicable)**	NA
Human tissue samples	NA
Data collected / Storage	NA
**KEY STUDY CONTACTS**	Full contact details including phone, email and fax numbers
Chief Investigator	Professor Gill Livingston<br>Professor of Psychiatry of Older People

	University College London<br>Division of Psychiatry<br>6th Floor Maple House (wing A)<br>149 Tottenham Court Road<br>London WC1 7TF<br>0207 679 9435<br>g.livingston@ucl.ac.uk

KEY ROLES AND RESPONSIBILITIES

SPONSOR: The sponsor is responsible for ensuring before a study begins that arrangements are in place for the research team to access resources and support to deliver the research as proposed and allocate responsibilities for the management, monitoring and reporting of the research. The Sponsor also has to be satisfied there is agreement on appropriate arrangements to record, report and review significant developments as the research proceeds, and approve any modifications to the design.

FUNDER: The funder is the entity that will provide the funds (financial support) for the conduction of the study. Funders are expected to provide assistance to any enquiry, audit or investigation related to the funded work. If further arrangements have been agreed with the funder, please refer to the funding agreement and insert

CHIEF INVESTIGATOR (CI): The person who takes overall responsibility for the design, conduct and reporting of a study. If the study involves researchers at more than once site, the CI takes on the primary responsibility whether or not he/she is an investigator at any particular site.

The CI role is to complete and to ensure that all relevant regulatory approvals are in place before the study begins. Ensure arrangements are in place for good study conduct, robust monitoring and reporting, including prompt reporting of incidents, this includes putting in place adequate training for study staff to conduct the study as per the protocol and relevant standards.

The Chief Investigator is responsible for submission of annual reports as required. The Chief Investigator will notify the RE of the end of the study, including the reasons for the premature termination. Within one year after the end of study, the Chief Investigator will submit a final report with the results, including any publications/abstracts to the REC.

PRINCIPLE INVESTIGATOR (PI): Individually or as leader of the researchers at a site; ensuring that the study is conducted as per the approved study protocol, and report/notify the relevant parties – this includes the CI of any breaches or incidents related to the study.

STUDY MANAGER: Organising the study as per the approved study protocol, liaising with study sites, participants and co-applicants, and report/notify the relevant parties – this includes the CI of any breaches or incidents related to the study

KEY WORDS depression, anxiety, third sector, minority ethnic groups, copings strategies

Family Carers, Dementia

LIST OF ABBREVIATIONS

Commonly used abbreviations – add or delete as applicable

AE	Adverse Event
AR	Adverse Reaction
CI	Chief Investigator
CRF	Case Report Form
CRO	Contract Research Organisation
DMC	Data Monitoring Committee
GAfREC	Governance Arrangement for NHS Research Ethics
HTA	Human Tissue Authority
IB	Investigator Brochure
ICF	Informed Consent Form
MD	Medical Device
ISRCTN	International Standard Randomised Controlled Studies Number
PI	Principle Investigator
PIS	Participant Information Sheet
QA	Quality Assurance
QC	Quality Control
RCT	Randomised Clinical Study
REC	Research Ethics committee
SAR	Serious Adverse Reaction
SAE	Serious Adverse Event
SDV	Source Data Verification
SOP	Standard Operating Procedure
SSI	Site Specific Information
TMF	Trial Master File

AS	Alzheimer’s Society
DA	Dementia Adviser
SA	South Asian
TS	Third Sector
ME	Minority Ethnic
CFIR	Consolidated Framework for Implementation Research
KTA	Knowledge to Action
RE-AIM	Reach, Efficacy, Adoption, Implementation, and Maintenance
START	Strategies for RelaTives
HADS	The Hospital Anxiety and Depression Scale
CSRI	Client Receipt Service Inventory
HSQ	Health Status Questionnaire

CONTENTS

1 INTRODUCTION ... 8
2 BACKGROUND AND RATIONALE ... 8
3 OBJECTIVES ... 9
3.1 Primary Objective ... 9
3.2 Secondary Objectives ... 10
4 STUDY DESIGN ... 10
5 STUDY SCHEDULE ... 12
6 CONSENT ... 14
7 ELIGIBILITY CRITERIA ... 15
7.1 Inclusion Criteria ... 15
7.2 Exclusion Criteria ... 15
8 RECRUITMENT ... 15
9 STATISTICAL METHODS ... 16
10 PATIENT AND PUBLIC INVOLVEMENT (PPI) ... 16
11 FUNDING AND SUPPLY OF EQUIPMENT ... 16
12 DATA HANDLING AND MANAGEMENT ... 17
13 MATERIAL/SAMPLE STORAGE ... 18
14 PEER AND REGULATORY REVIEW ... 18
15 ASSESEMENT AND MANAGEMENT OF RISK ... 18
16 RECORDING AND REPORTING OF EVENTS AND INCIDENTS ... 19
16.1 Definitions of Adverse Events ... 19
16.2 Assessments of Adverse Events ... 19
16.3 Recording adverse events ... 21
16.4 Procedures for recording and reporting Serious Adverse Events ... 21
16.5 Serious Adverse Events that do not require reporting ... 23
16.6 Reporting Urgent Safety Measures ... 23
16.7 Protocol deviations and notification of protocol violations ... 23
16.8 Reporting incidents involving a medical device(s) (if applicable) ... 23
16.9 Trust incidents and near misses ... 23
17 MONITORING AND AUDITING ... 24

18 TRAINING 24
19 INTELLECTUAL PROPERTY 24
20 INDEMNITY ARRANGEMENTS 24
21 ARCHIVING 24
22 PUBLICATION AND DISSEMINATION POLICY 25
23 REFERENCES 25
24 APPENDICES 27

1 INTRODUCTION

Family members providing care to a person with dementia are often distressed, anxious or depressed. We developed the START (**Stra**tegies for **R**ela**T**ives) intervention for family carers of relatives with dementia to promote helpful coping strategies for the difficulties of caring, and reduce these types of symptoms. The original START intervention was delivered within NHS service structures by psychology graduates. The main purpose of this study is to widen access to the START intervention by adapting it for delivery in the third sector and to Minority Ethnic (ME) groups, within existing structures of the Alzheimer's Society (AS) and the South Asian community in the first instance. The START intervention and materials will be adapted in **Phase 1** of the study (see Figure 1). In **Phase 2** we will implement the modified intervention, within existing structures of the Alzheimer's Society and the South Asian community respectively. In **Phase 3**, an independent evaluation will assess the impact of the intervention incorporating qualitative and quantitative information. Benefits are expected in terms of improved access for family carers including those in hard to reach groups to START, as well as a template for implementation of the START intervention in the third sector and for other ME groups.

Figure 1 Study flowchart

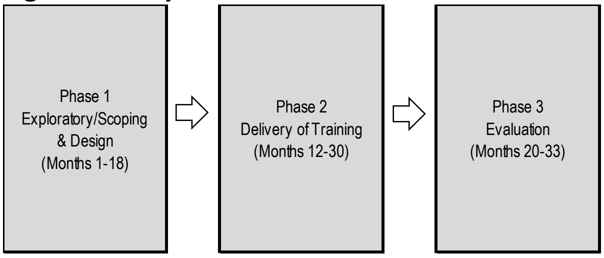

2 BACKGROUND AND RATIONALE

Family carers of people with dementia experience higher levels of psychological burden than carers of other people with chronic illness (1, 2), with up to 40% showing clinical levels of depression and anxiety (3, 4). Maintaining the health of family carers of people with dementia is socially and economically important (5, 6) as it may prevent or delay care home entry by improving family carers wellbeing so they can continue caring (5).

The START intervention

We developed the START (Strategies for RelaTives) (7) intervention to help family carers manage their own symptoms of depression and anxiety, to better cope with caring for a relative with dementia. START is an eight-session manual-based coping intervention for family carers delivered by trained psychology graduates, who are supervised by clinical psychologist. The START sessions comprise: behavioural management, communication strategies, identifying and changing unhelpful thoughts, accessing emotional support, increasing pleasant events, relaxation, future planning and developing a maintenance plan. The intervention also includes home practice between sessions, using the manual and a stress reduction CD (7). START is effective at improving carers' mood and anxiety levels at 8 months and 2 years (8, 9), and shows continuing benefits at 6 years (paper in preparation). It is also cost-effective when compared to benefit in the range the government judges worth funding (9).

START in the third sector

While our dissemination work has resulted in START's wider availability, this has not yet been optimised for varied reasons. These include lack of money, available staff and fit with existing NHS

service structures (10). In places where START has been implemented there are waiting lists for the treatment because of the huge number of carers who may potentially benefit. It is important to consider how to support more carers and to build upon existing third sector infrastructure, as many prefer interventions in a non-health setting. If availability were extended, including to the third sector, then many more carers could benefit. Although the AS currently deliver complementary services to carers and a wealth of experience and knowledge exists in the third sector, non-evidence based interventions often dominate. Developing pathways for START delivery in the third sector would help bridge this gap.

START for Minority Ethnic (ME) groups and the South Asian community
Although the START intervention and manual have been standardised and made available online with a training programme for trainers (see http://www.ucl.ac.uk/psychiatry/start), they have not been adapted or tested with different ethnicities and cultures. Minority ethnic (ME) people have specific needs related to culturally different understanding of dementia and consequent late presentation, which we have detailed in our earlier qualitative study (11) Barriers to help-seeking for dementia in ME groups vary greatly (12-15) and include negative experiences and feelings about services, stigma, and reluctance to reveal private or sensitive information, share or relinquish family care to people outside the family circle (16-18). These may mean that ME people miss out on support provided by services and feel isolated due to lack of awareness of service availability and appropriateness to their culture (19). By the time people contact services, people living with dementia may have higher care needs and families may already be coping with severe behavioural changes. Thus, ethnicity and culture have a unique relationship with the dementia care pathway, and affect how patients and carers respond to health interventions.

In this project, we will build the foundations to adapt the START intervention and manual for the South Asian population in the UK. Alzheimer's Society have successfully delivered a four session programme about dementia to South Asian carers (Information Programme for South Asian Families) through bilingual workers. Our Bradford collaborators' evaluation of this programme found that community partners with established reputations and in-depth knowledge about culturally acceptable and effective recruitment strategies played an essential role (20). START is flexible and has different components, with diverse strategies that enable it to be individually tailored to carer circumstances and changing needs. There may be the need for cognitive reframing of the intervention, emphasising the benefit to the person with dementia, rather than a relinquishing of responsibility or prioritising the carer's emotional needs (21). Consideration of pleasant, stress-relieving activities may need to consider the wider family support network, as well as support from local religious and community organisations (13). Furthermore, as the people living with dementia present later, different symptoms have to be considered and the balance of time devoted to initial orientation and discussion may need to shift in favour of more targeted coping strategies. The intervention may require more emphasis on rights and financial benefits to respond to greater socio-economic need for some, and to enable carers to be assertive with care providers if their needs are not taken seriously due to racism. Moreover, there are sometimes, but not always, language barriers that may need to be addressed with the use of bilingual facilitators.

3 OBJECTIVES

3.1 Primary Objectives

To lay the foundations for widening access by

- Providing a framework for implementing and delivering START in the third sector.
- Adapting START for Minority Ethnic (ME) communities, starting with the exemplar of the South Asian community.

3.2 Secondary Objective

Provide a template for both the third sector and other minority ethnic groups facing similar barriers to deliver the START intervention

4 STUDY DESIGN

This is a mixed methods study designed to assess implementation of a modified START intervention for family carers of relatives with dementia, within existing structures of the Alzheimer's Society and the South Asian community. The total study duration is 36 months from study set up through to write up (see Figure 2). The study will be carried out in three phases, with some overlap between phases.

4.1.1 Phase 1

Our first implementation framework will be used prior to beginning implementation to describe and guide the process (22). We will use the widely employed Consolidated Framework for Implementation Research tool (CFIR) (22, 23), a structured tool with five domains, divided into a total of 35 defined constructs, to ensure the team systematically assesses potential barriers and facilitators in preparation for implementation. The CFIR provides a pragmatic structure for approaching complex problems of implementation. We will, as recommended, start by explicitly selecting and justifying which of the CFIR constructs we will use (22, 23).

We will undertake focus groups or individual interviews as appropriate and consult with Alzheimer's Society stakeholders and family carers of relatives with dementia from South Asian backgrounds (using translators as appropriate), to inform changes to the START intervention.

Consent procedures
Informed consent will be sought from all participants taking part. Participants will be informed that all data collected will be anonymised and that their personal information will be kept confidential although the data will be used for research. We will ask for the participants' permission to audio-record the focus groups and individual in-depth interviews, and inform them that the audio tapes will be destroyed at the end of the study. Participants will be informed that they can withdraw their consent at any time. In seeking consent, we will follow the Mental Capacity Act (2005) provisions when deciding whether to include people and will not include those who do not have capacity. Trained mental health researchers will conduct the interviews. We will use focus groups (for Alzheimer's Society staff and South Asian carers) or individual interviews as appropriate. If the participant becomes upset or distressed, we will offer to stop the interview and check that they are willing to proceed before continuing with the interview.

Training and delivery
Our project researchers are psychology graduates. They have a degree in psychology but without clinical psychology training. They have been recruited on evidence of listening skills, empathy and dementia experience and have experience in qualitative work and have attended training and are supervised in their work. This level of expertise will ensure breadth of skills. A short training programme will be delivered by our leads. Project researchers have all completed UCL training in safety and diversity and training in Good Clinical Practice and informed consent. They will also have teaching sessions dedicated to cultural sensitivity. Training will emphasise the need to operate from an inclusive values base and to respect diversity and the existing knowledge and experience of those they interview.

We will then use a process model, the Knowledge-to-Action (KTA) Framework (24) to design a practical strategy for translating our findings into practice, incorporating what we have learned of the context and situation in which we are implementing through the CFIR (22). The KTA Framework

is a process intended to guide the process of translating research into practice and has an emphasis on stakeholder involvement and tailoring knowledge to the needs of those who are going to use it. The Framework contains two distinct but mutually influencing components, one focused on Knowledge Creation and one called the Action Cycle. The Action Cycle reflects the practical actions needed to apply knowledge in practice. The Framework includes consideration of how to adapt knowledge to local context, assessment of barriers to knowledge use, and tailoring of interventions – we will be doing this in detail through our use of CFIR in the first phase. As the cyclical nature of the Framework suggests, our work to explore and understand context using CFIR and to ensure effective implementation through the KTA Framework will involve iterative elements rather than a simple linear process.

We will scope the training needs of the potential Alzheimer's Society and South Asian therapists as they will have different training and support needs than the psychology graduates we had for START. We will make changes to the training manual and START manuals for the third sector as needed. We will change the START manuals (carer and therapist version) words and illustrations using the knowledge generated to fit with South Asian culture and time of presentation. We will also produce training materials. We will then consult with people whom we have interviewed in the Knowledge to Action phase to refine it further. We will employ translators to translate and back-translate the manuals from English to Punjabi as the most common South Asian language. We will employ translators to "smooth out" inaccuracies iteratively until it is correct.

Changes will result in a modified START intervention for the Third Sector (TS) **"RE START TS"**, and for the South Asian (SA) Community **"RE START SA"**.

4.1.2 Phase 2

In Phase 2 (month 12 to 30) we will train three (n=3) Alzheimer's Society Dementia Advisers (DA) to deliver RE START TS to nine (n=9) family carers of relatives with dementia from the Alzheimer's Society, and project researchers/bilingual facilitators to deliver RE START SA to three (n=3) South Asian carers in Punjabi, and to between eight and ten (n=10) South Asian carers in English.

4.1.3 Phase 3

In Phase 3 (month 20 to 33) Iain Lang will lead evaluation to assess the impact of the intervention incorporating qualitative and quantitative information covering the fields of the MRC process evaluation framework (25). He will use the RE-AIM implementation tool (or best suited equivalent) which specifies the aspects of implementation that should be evaluated specifically:

- Reach (rate of acceptance and demographics of those taking it up, participants' views of experience, unexpected and expected),
- Effectiveness (dose – number of sessions delivered to individuals; fidelity – using session recording; baseline and post-intervention mood – The Hospital Anxiety and Depression scale(HADS) (26, 27), and Quality of Life, Health Status Questionnaire (HSQ) (26, 28) as in START(7),
- Adoption (Developing diverse partnerships)
- Implementation (training documents and manuals for further implementation)
- Maintenance (embedding in long term infrastructure)

RE-AIM (29) has previously been used in the USA in similar projects (30). Evaluation will be independent: the researcher who delivers the intervention will not be the one who evaluates. Although there will be summative aspects to this evaluation, in an important sense it will be formative, in that it will inform subsequent implementation and spread of START. We also anticipate that what we will learn things from this project about how to implement evidence-based practice in dementia care in diverse community settings in ways that go beyond the specifics of START.

To promote sustainability beyond the end of the grant, we will revisit implementation sites towards the end of the project to assess the sustainability of our implementation work. We have engaged an NHS trust medical director as co-applicant and the London dementia network as collaborators. We will publish our work in peer reviewed journals, present it at conferences and work with the AS to publicise it. The AS is committed to promoting it in the voluntary sector if successful, by holding workshops for voluntary sector providers and commissioners to learn about START and access resources.

Figure 2 GANTT chart

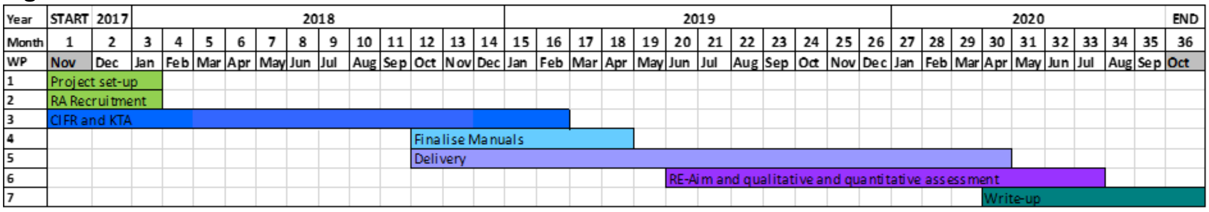

<u>Participant groups and sampling procedure</u>

We will use a purposeful sampling technique widely used in qualitative research for the identification and selection of information rich cases for the most effective use of limited resources, and to achieve a depth of understanding (31). We will sample participants to capture maximum variation in sociodemographic characteristics, and other variables including time in post at the Alzheimer's Society, relationship to relative with dementia (e.g. spouse or child) and living situation. We will obtain a comprehensive understanding by continuing to sample until no new substantive information is acquired (theoretical saturation). We will collect socio-demographic information on all participants included in the study. The interviews will be audio tape-recorded and transcribed verbatim. We will use a topic guide derived from the literature which will develop further during the interviews in an iterative fashion (32). The data will be qualitatively analysed using thematic analysis to identify broad similarities and differences in the data.

Phase 1 participant groups will inform the adaptation of the START intervention and materials, and include (i) up to ten (n=10) Alzheimer's Society nominated Dementia Advisers, (ii) up to ten (n=10) Alzheimer's Society nominated managers and (iii) up to twenty (n=20) family carers of relatives with dementia from South Asian backgrounds. Phase 2 participant groups include (i) three (n=3) Alzheimer's Society nominated Dementia Advisers who will be trained to deliver the RE START TS intervention to (ii) up to nine (n=9) Alzheimer's Society nominated carers and (iii) up to thirteen (n=13) family carers of relatives with dementia from South Asian backgrounds who will receive the RE START SA intervention, delivered by project researchers in English or a South Asian language.

<u>Enrolment (duration) and follow up</u>

Participants recruited to Phase 1 of the study will be enrolled from consent through to finalisation of the manuals at month 16, i.e. approximately 13 months including follow up (i.e. consultation on changes to the intervention). Participants recruited to Phase 2 will be enrolled from consent through to completion of the evaluation at month 33, i.e. approximately 22 months including follow up (i.e. post intervention quantitative measures and interviews). Participants can withdraw from the study at any time, and data collected up to this point will be included. This will be recorded using an exit form.

5 STUDY SCHEDULE

Recruitment will commence subject to necessary approvals, following procedures described in section 8 of this document. We will obtain informed consent from all participants including

Alzheimer's Society staff and family carers of relatives with dementia, following procedures described in section 6 of this document.

In **Phase 1** (See Figure 3), we will interview Alzheimer's Society stakeholders (i.e. Dementia Advisers and Managers nominated by the Alzheimer's Society) and family carers from South Asian backgrounds about the START intervention using the Consolidated Framework for Implementation Research Tool (CFIR) (22, 23) to assess baseline barriers and facilitators, and scope voluntary sector and bilingual facilitators' training and support needs. Participants will be interviewed following consent at a location convenient to them or at UCL. The Knowledge-to-Action framework (KTA) (24) will be used as a guide for translating these findings into practice, i.e., designing the RE START TS, RE START SA interventions and materials. Work to explore and understand context using the CFIR and to ensure effective implementation through the KTA framework will involve iterative elements rather than a simple linear process. We will follow up with Phase 1 interviewees to consult on changes and further refine interventions and materials.

In **Phase 2**, we will (i) train and supervise AS Dementia Advisers to deliver RE START TS to AS nominated family carers and (ii) train and supervise researchers and bilingual facilitators to deliver RE START SA to family carers from South Asian backgrounds in English or in a South Asian Language. It is difficult to provide extensive detail regarding training, supervision and delivery procedures as these will be determined in large part by Phase 1 results. However, procedures are likely to be some variation of those followed in previous studies in which START has been delivered (7).

The original START intervention is designed to be delivered in eight sessions over 8-14 weeks. The intervention is delivered where the carer prefers, usually at their home. The sessions cover (1) Introduction: Learning about dementia, carer stress and understanding behaviours of the person cared for; (2−6) Discussion of problems that the carer finds difficult, incorporating behavioural management techniques; skills to take better care of themselves, including changing unhelpful thoughts, assertive communication, relaxation and planning pleasant activities; increasing communication; promoting acceptance; where to get emotional support and positive reframing; (7) Future needs of the patient, with psychoeducation about care and legal planning, specifically adapted to the UK; (8) Maintaining the skills learned over time. Carers are given homework tasks to complete between sessions, including identifying triggers and reactions to challenging behaviours, and identifying and challenging negative thoughts.

Training of Dementia Advisers and researchers will likely be delivered over two (2) days and contain a strong practical focus on how to deliver the intervention, potential clinical dilemmas, empathic listening skills, effective use of supervision, safe working practice and when to ask for help. We will train participants to adhere to the manual; they will be asked to demonstrate, by role-play, competence in delivering each session of the intervention. Following training, formal clinical supervision will begin. Our clinical psychologist, PR, will likely meet with Dementia Advisers and researchers separately for 1.5 hours of group supervision a fortnight. In addition to this group supervision, PR will be available for individual supervision. The supervision format will be tailored to reflect the specific needs of the research project. Supervision will perform a number of functions including case management, clinical skills development, monitoring the fidelity to the manualised intervention, ensuring safe practice with clients and staff support. Once Dementia Advisers (DSWs) are fully trained, they will hold a caseload of three (3) family carers for the intervention. Researchers will hold a caseload of approximately 4-6 family carers each.

In **Phase 3**, Iain Lang (University of Exeter) will lead the evaluation will use the RE-AIM (Reach, Effectiveness, Adoption, Implementation, and Maintenance) implementation framework (29) (or best suited equivalent) to assess the impact of the intervention incorporating qualitative and

quantitative information. To monitor fidelity to the manualised intervention, facilitators will record one session per family carer. Quantitative measures (see below) will be administered to carers prior to the intervention, immediately after the intervention and at one year. Follow up with Phase 2 participants will also include interviews about the intervention immediately after using the RE-AIM evaluation framework as a guide.

We will use validated standardised quantitative measures to inform the evaluation (and see if the changes are those expected from the original study) administered to carers prior to the intervention, i.e., at Baseline (B), immediately after the intervention (T1) and at one year (T2). Quantitative measures will include:

(i) The Hospital Anxiety and Depression Scale (HADS) (26, 27), a validated, reliable measure to measure mood in carers.

(ii) The carer Health Status Questionnaire (HSQ) (28), a 12 item health-related quality of life scale.

(iii) The widely used Client Receipt Services Inventory (CSRI) (33), to measure the service use consequences for the carer and the relative with dementia.

Figure 3 Study phases, theoretical frameworks and procedures

Study Phases	Implementation Framework	**RE-START TS** (Third Sector)	**RE-START SA** (South Asian ME Group)
Phase 1<br>Exploratory/Scoping & Design<br>(Months 1-18)	**C**onsolidated **F**ramework for **I**mplementation **R**esearch tool (CFIR) / Knowledge-to-Action (KTA)	Phase 1 **qualitative interviews** with Alzheimer's Society Dementia advisers and Managers<br><br>**Scoping** AS Dementia Adviser training needs<br><br>**Design** RE START TS Package (manuals, training materials)<br><br>**Consult** with Phase 1 interviewees on changes	Phase 1 **qualitative interviews** (South Asian Family Carers)<br><br>**Scoping** Blingual Facilitator needs<br><br>**Design** RE START SA Package (manuals, training materials)<br><br>**Translate and Record** RE START SA Package<br><br>**Consult** with Phase 1 interviewees on changes
Phase 2<br>Delivery of Training<br>(Months 12-30)	(**R**each, **E**ffectiveness, **A**doption, **I**mplementation, and **M**aintenance)<br>RE-AIM Implementation Tool	**Administer** pre-training quantitative measures (CSRI, HADS, HSQ) - Baseline<br><br>**Train** three (3) AS Dementia Adviser to deliver RE START TS<br><br>**Supervise** delivery of RE START TS to nine (9) AS carers	**Administer** pre-training quantitative measures (CSRI, HADS, HSQ) - Baseline<br><br>**Train** Bilingual Facilitators to deliver RE START SA<br><br>**Deliver** RE START SA in South Asian Language to three (3) South Asian carers<br><br>**Deliver** RE START SA in English to btwn eight and ten (8-10) South Asian carers
Phase 3<br>Evaluation<br>(Months 20-33)		**Administer** post-training quantitative measures (CSRI, HADS, HSQ) - T1 and T2<br><br>Post-training **qualitative interviews** (AS Dementia Advisers and Family Carers)	**Administer** post-training quantitative measures (CSRI, HADS, HSQ) - T1 and T2<br><br>Post-training **qualitative interviews** (Bilingual Facilitators and South Asian Family Carers)

6 CONSENT

Members of the research team will obtain individual informed consent from all participants including Alzheimer's Society staff and family carers of relatives with dementia, at a location convenient to them. Prospective participants who have agreed to participate in the study will be asked to sign a consent form prior to data collection. Potential participants will be given no less than

24 hours from receipt of the participant information sheet, before being contacted by a member of the research team to consent or not to take part. In seeking consent, we will follow the Mental Capacity Act (2005) provisions when deciding whether to include people and will not include those who do not have capacity. Participants will be informed that all data collected will be anonymised and that their personal information will be kept confidential according to the Data Protection Act and according to UCL information governance procedures, although the data will be used for research. We will ask for participants' permission to audio-record focus groups and semi-structured interviews, and at least one (1) training session. We will make clear that audio data files will be destroyed at the end of the study. Participants will be informed that they can withdraw consent at any time without being obliged to provide a reason. If one is offered, it will be recorded by the research team. Non-native English speaking family carers of relatives with dementia will be approached by a bilingual member of the research team and/or a member of the research team accompanied by a translator.

7 ELIGIBILITY CRITERIA

7.1 Inclusion Criteria

Family carers of relatives with dementia need to be 18 years or over, providing care or support (or having done so in the past) to a relative with dementia and willing to provide informed consent to participate in the study

Alzheimer's Society Dementia Advisers and Managers need to be 18 years or over and willing to provide informed consent to participate in the study.

Dementia Advisers must be currently employed by the Alzheimer's Society actively fulfilling their role.

7.2 Exclusion Criteria

None

8 RECRUITMENT

Dementia Advisers in both phases will be identified and nominated by the Alzheimer's Society, as will managers and carers. Family carers of relatives with dementia from South Asian backgrounds will be identified and approached via the London Dementia Network, Camden and Islington NHS Foundation Trust Memory Services, and Bradford based community organisations with whom co-applicant Kathryn Lord has collaborated with in previous studies.

The Alzheimer's Society will provide nominated staff and carers with Participant Information Sheets, and refer prospective participants to the research team. The research team will make contact no less than 24H later, provide further details regarding the study if needed and extend an invitation to participate in the study.

In the case of family carers of relatives with dementia from South Asian backgrounds, some prospective participants may have already agreed to be contacted for research (as trusts may have a register), but we will still approach a clinician beforehand to ensure there have been no changes and ask, if possible, if they can initiate contact. Clinicians or social workers will provide prospective participants with Participant Information Sheets (PIS) detailing the purpose of the study and what participation in the study entails, and obtain verbal consent and/or confirmation for referral to the research team. Participant Identification Centres will be asked to verbally provide potential

participants details. In cases where written information is provided in hard copy or electronically, these will be stored following procedures outlined above, and any emails permanently deleted immediately after. The research team will make contact no less than 24H later, provide further details regarding the study if needed and extend an invitation to participate in the study. Members of the research team will obtain informed consent from all participants that have agreed, following procedures described in section 6 of this document.

9 STATISTICAL METHODS

This is an implementation study not designed to assess clinical and cost effectiveness of START which is already established. Quantitative measures will be summarised simply using descriptive statistics. The study does not necessitate any other statistical considerations.

10 PATIENT AND PUBLIC INVOLVEMENT (PPI)

We will ensure public and patient involvement (PPI) throughout the research and implementation. We consulted the Alzheimer's Society Research network (ASRN) on the project development and methodology. Shirley Nurock (co-applicant) will lead PPI. We will ensure we have south Asian carer representation and ask members of the ASRN to advise throughout, including on the steering and project management committee. We would review the AS's role in supporting wider-implementation of START with the research team in the final year of the grant.

11 FUNDING AND SUPPLY OF EQUIPMENT

The study funding has been reviewed by the UCL Research Office, and deemed sufficient to cover the requirements of the study. The research costs for the study have been supported by The Alzheimer's Society with a grant of £397,517.17 awarded in August 2017.

The project manager, will work for the three years organising the whole project, leading the ethics and local submission, recruiting and interviewing, beginning and co-ordinating the CFIR completion, being one of two coders of qualitative interviews, co-ordinating the new manuals completion, delivering the intervention to some of the English-speaking South Asian carers, writing up and line managing the research assistant (total cost £145,413.97). The RA will be employed for 2. 25years from February 2018 at grade 6B gathering and analysing data, and delivering some of the interventions phase. The manager and RA will each require a computer (cost £724.53 each). We estimate we will require 40 qualitative interviews and plan to give participants £30 vouchers as a token for their time at a cost of £1,200. We will need two digital recorders for the qualitative interviews (Total £147.82). For safety and so participants can reach them both researchers will have a mobile phone. Costs are £20/month plus VAT; a total this will be 60 months allowing for inflation over the three years (487.96) We will pay Way with Words for interviews transcription £1.20 + VAT per minute of individual audio-recording. We estimate that individual interviews will be an hour and focus groups take longer to transcribe - 40 would cost £4,554.66 in total. Translation costs are £40+VAT per hour of interpreter time or around £200 to translate roughly 1.5 pages of A4 text. Currently the leader and carer version are each 173 pages long but as there are many pictures and the text is spread out we estimate that this is equivalent to about 86 pages so translation should be £5,733, with back translation the same and then some smoothing out at around £2,000. We also estimate we will need 20 hours of interpreters for the qualitative interviews = £960 plus VAT. Total translation cost including VAT £17,624.05. We have costed for one session (half a day) of Naaheed Mukadam's time from January 2018 for her expertise in cross cultural work (prior to that from NIHR grant; total £24,115.71). We have also costed for one day a week of Dr Rapaport's time on the

project to take a lead part in producing the new manuals, and to train therapists in the intervention and supervise its delivery (total £63,562.92) We have also costed for 2 hours of Andrew Sommerlad's time from May 2019 to lead on qualitative interviews (prior to that from Wellcome fellowship. Total £5,366.78). We have costed Kathryn Lord's salary at 2 hours a week after the end of her Bradford fellowship (£4,127) to liaise between UCL and Bradford and interview in Bradford. We have costed for £6000 to pay for interviewing, training, intervention delivery by and supervision of three Dementia Advisers from the AS in discussion with the AS. This is at £12.94 per hour = 463 hour. This encompasses an estimated 50 hours of interviews and travel before and after the intervention about barriers, facilitators and how it worked, 120 hours of training/ travel to and from training, 30 hours to recruit the participants, 156 hours to deliver intervention an/travel to people's home, 10 hours of phone calls to make arrangements, 28 hours of write up about sessions, 70 hours of supervision/ travel. The cost of training the bilingual worker and them delivering the intervention will be similar. The cost of training the bilingual worker and them delivering the intervention will be similar.

We have costed for 12 return travel between Bradford and London at £138 return. Kathryn Lord will visit London or our team will visit Bradford every three months, to interview people from South Asian community or to or liaise about the project. We have costed for 12 return trips at £150 for off peak fares for Iain Lang to come to project meetings and advise and supervise on the evaluation We have also budgeted travel for the researchers to interview and deliver the intervention, assuming an average of £10 per return visit (total £6000) We expect to have three steering group meetings and eight project group meetings over the life of the project and have budgeted £3,817.28 for meeting costs, including lunch and local travel (as it is cheaper for travel to be off peak). We have budgeted for 2 PPI members to attend the steering and management meetings at £50 per meeting (£1,200). We have budgeted £150 for stationery as we have to write to participants to confirm interviews and provide manuals.

12 DATA HANDLING AND MANAGEMENT

This project has been registered with the UCL Data Protection Team (registration number: Z6364106/2018/01/57). All information gathered will be kept strictly confidential and held in accordance with the principles of the Data Protection Act (1998), and according to UCL information governance procedures. Alzheimer's Society staff will work in accordance with the Alzheimer's Society data handling and management procedures.

Hard copy data of participant consent forms will be stored at the Division of Psychiatry, UCL, in a secure locked location accessible only by researchers.

Each participant will be assigned a unique identifying number the key of which will be kept electronically in the study's UCL shared drive accessible only by the UCL research team. All data will be stored without subject name or contact details. Data will be held on a secure database on a password-protected computer at University College London. Access to data will be restricted to appropriate members of the research team. In order to enable follow-up contacts, it will be necessary to identify the participants, but access to contact details (e.g. name and address) will be restricted to key members of the research team.

Participants will be informed how data will be managed as part of the consent procedure. Rules governing the conduct of the focus groups will be discussed at the beginning of each group and participants will be requested not to discuss others' contributions outside the group.

Audio recordings from focus groups/interviews and training sessions will be uploaded to the UCL Data Safe Haven as soon as possible after they are acquired. Once uploaded recordings will be

deleted from recording devices. Audio files will be sent to a professional UK-based commercial transcription service (WAY with Words) that has strong data protection, confidential storage and removal from the transcription service database when complete. After data processing the data will be archived and stored securely in accordance with UCL protocols.

Co-applicants Kathryn Lord and Iain Lang will act as custodians of data at the University of Bradford and the University of Exeter respectively, who are required by law to comply with the data protection Act, 1998. No identifiable data will be transferred between study sites (i.e. London, Bradford and Exeter Universities) over the course of the study. In order to ensure security of anonymised data during transfer of data between study sites, data will be transferred via University Email accounts only, downloaded and stored following procedures outlined above, and permanently deleted from electronic inboxes immediately after.

Participant Identification Centres will be asked to verbally provide potential participants details. In cases where written information is provided in hard copy or electronically, these will be stored following procedures outlined above, and any emails permanently deleted immediately after.

13 MATERIAL/SAMPLE STORAGE

Not applicable

14 PEER AND REGULATORY REVIEW

The study has been peer reviewed in accordance with the requirements outlined by UCL.

Choose either (having discussed with the UCL/UCLH Research Office):

- The Sponsor considers the procedure for obtaining funding from the Alzheimer's Society to be of sufficient rigour and independence to be considered an adequate peer review.

The study was deemed to require regulatory approval from the following bodies: Health Research Authority Research Ethics Committee. Approval will be obtained before the study commences.

15 ASSESMENT AND MANAGEMENT OF RISK

In delivering the intervention to family carers of relatives with dementia, Dementia Advisers will work according to the Alzheimer's Society's Risk Management policies. An important aspect of supervision of the researchers delivering the intervention to family carers from South Asian backgrounds will be to identify and respond to any risks identified during the course of assessment or intervention. Researchers will be provided with specific training in how to respond to any risks disclosed by carers, in relation to harm to both themselves and the person they were caring for. Researchers will be asked to speak to the lead investigator if the abuse Modified Conflict Tactic Score (MCTS) (34, 35) is in the possibly abusive range (≥ 2) or if they had any concerns about potential abuse. Similarly, if carers reported high levels of depression or anxiety, this will be discussed with the lead investigator. If concerns are raised, a plan will be made with the lead investigator about how to manage the risk and information will be shared with the local clinical teams. Time will also be taken within supervision to highlight the importance of behaving ethically and safely in all aspects of clinical work, for example reflecting on maintaining clear boundaries

clinically and how to practise safely when working alone in people's homes .Researchers will work to UCL's Lone Working standard in cases where they visit the family carer at their home.

16 RECORDING AND REPORTING OF EVENTS AND INCIDENTS

16.1 Definitions of Adverse Events

Term	Definition
Adverse Event (AE)	Any untoward medical occurrence in a patient or study participant, which does not necessarily have a causal relationship with the procedure involved.
Serious Adverse Event (SAE).	Any adverse event that:<br>• results in death,<br>• is life-threatening*,<br>• requires hospitalisation or prolongation of existing hospitalisation**,<br>• results in persistent or significant disability or incapacity, or<br>• consists of a congenital anomaly or birth defect
*A life- threatening event, this refers to an event in which the participant was at risk of death at the time of the event; it does not refer to an event which hypothetically might have caused death if it were more severe.<br>** Hospitalisation is defined as an in-patient admission, regardless of length of stay. Hospitalisation for pre-existing conditions, including elective procedures do not constitute an SAE.	

16.2 Assessments of Adverse Events

Each adverse event will be assessed for severity, causality, seriousness and expectedness as described below.

16.2.1 Severity

The generic categories below are given for use as a guide. You may have a more specific scale that you want to use related to the disease (e.g. CTCAE criteria), (amend as required).

Category	Definition
Mild	The adverse event does not interfere with the participant's daily routine, and does not require further procedure; it causes slight discomfort
Moderate	The adverse event interferes with some aspects of the participant's routine, or requires further procedure, but is not damaging to health; it causes moderate discomfort
Severe	The adverse event results in alteration, discomfort or disability which is clearly damaging to health

16.2.2 Causality

The assessment of relationship of adverse events to the procedure is a clinical decision based on all available information at the time of the completion of the case report form.

If a differentiated causality assessment which includes other factors in the study is deemed appropriate, please add/amend the following wording to specify:

It is of particular importance in this study to capture events related to the product application procedure (specify e.g. surgery) / product failure / mandatory concomitant medications (specify e.g. conditioning chemotherapy). The assessment of relationship of an adverse event to this/these additional safety issue(s) will also be carried out as part of the study.

The differentiated causality assessments will be captured in the study specific CRF/AE Log and/or SAE form (amend as required).

The following categories will be used to define the causality of the adverse event:

**Category**	**Definition**
Definitely:	There is clear evidence to suggest a causal relationship, and other possible contributing factors can be ruled out.
Probably:	There is evidence to suggest a causal relationship, and the influence of other factors is unlikely
Possibly	There is some evidence to suggest a causal relationship (e.g. the event occurred within a reasonable time after administration of the study procedure). However, the influence of other factors may have contributed to the event (e.g. the participant's clinical condition, other concomitant events).
Unlikely	There is little evidence to suggest there is a causal relationship (e.g. the event did not occur within a reasonable time after administration of the study procedure). There is another reasonable explanation for the event (e.g. the participant's clinical condition).
Not related	There is no evidence of any causal relationship.
Not Assessable	Unable to assess on information available.

16.2.3 Expectedness

Category	Definition
Expected	An adverse event which is consistent with the information about the procedure listed in the Investigator Brochure, SPC, manual of Operation (amend as appropriate) **or clearly defined in this protocol.**

Unexpected	An adverse event which is not consistent with the information about the procedure listed in the manual of operation (or other – amend as appropriate)* **or clearly defined in this protocol.**

* this includes listed events that are more frequently reported or more severe than previously reported

16.3 Recording adverse events

Adverse events occurring during the period that the participant is registered with the study will be reported to the Sponsor immediately upon knowledge of the event (Research-incidents@ucl.ac.uk).

16.4 Procedures for recording and reporting Serious Adverse Events

All serious adverse events will be recorded in the medical records and the CRF, and the sponsor's AE log.

All SAEs (except those specified in section 16.5 as not requiring reporting to the Sponsor) must be recorded on a serious adverse event (SAE) form. The CI/PI or designated individual will complete an SAE form and the form will be preferably emailed to the Sponsor within 5 working days of becoming aware of the event. The Chief or Principal Investigator will respond to any SAE queries raised by the sponsor as soon as possible.

Where the event is unexpected and thought to be related to the procedure this must be reported by the Investigator to the Health Research Authority within 15 days.

Completed forms for unexpected SAES must be sent within 5 working days of becoming aware of the event to the Sponsor

Email forms to

Research-incidents@ucl.ac.uk (if sponsored by UCL)

Flow Chart for SAE reporting (this simple flow chart is for single site study, please amend in line with study specific requirements)

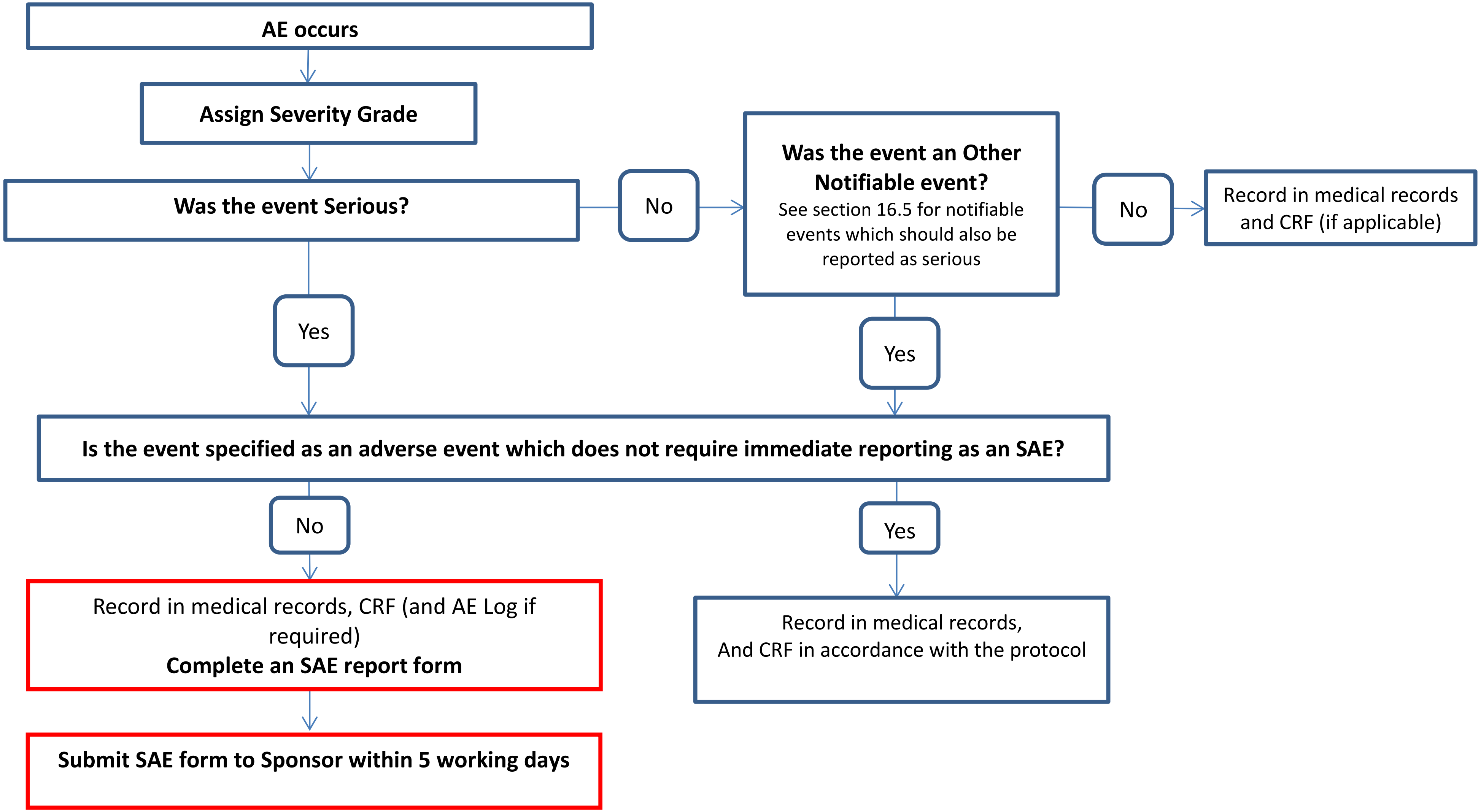

16.5 Serious Adverse Events that do not require reporting

You may choose not to report some particular SAEs to the sponsor, for example if they are expected to occur on a regular basis and offer no further new information to your safety profile or are related to the disease area of the participants. It should be specified that where the frequency or severity of these events is unusual they must be reported. These events must continue to be recorded in the medical records, CRF and the AE log (if required), however you may state that you will not complete an SAE form and forward it to the sponsor. Provide the rationale for doing so.

16.6 Reporting Urgent Safety Measures

If any urgent safety measures are taken the CI/ PI shall immediately and in any event no later than 3 days from the date the measures are taken, give written notice to the relevant REC and Sponsor of the measures taken and the circumstances giving rise to those measures.

16.7 Protocol deviations and notification of protocol violations

A deviation is usually an unintended departure from the expected conduct of the study protocol/SOPs, which does not need to be reported to the sponsor. The CI will monitor protocol deviations.

A protocol violation is a breach which is likely to effect to a significant degree –

(a) the safety or physical or mental integrity of the participants of the study; or
(b) the scientific value of the study.

The CI and sponsor will be notified immediately of any case where the above definition applies during the study conduct phase.

16.8 Reporting incidents involving a medical device(s) (if applicable)

Not applicable

16.9 Trust incidents and near misses

An incident or near miss is any unintended or unexpected event that could have or did lead to harm, loss or damage that contains one or more of the following components:

a. It is an accident or other incident which results in injury or ill health.
b. It is contrary to specified or expected standard of patient care or service.
c. It places patients, staff members, visitors, contractors or members of the public at unnecessary risk.
d. It puts the Trust in an adverse position with potential loss of reputation.
e. It puts Trust property or assets in an adverse position or at risk.

Incidents and near misses must be reported to the Trust through DATIX as soon as the individual becomes aware of them.
A reportable incident is any unintended or unexpected event that could have or did lead to harm, loss or damage that contains one or more of the following components:

a) It is an accident or other incident which results in injury or ill health.
b) It is contrary to specified or expected standard of patient care or service.

c) It places patients, staff members, visitors, contractors or members of the public at unnecessary risk.
d) It puts the Trust in an adverse position with potential loss of reputation.
e) It puts Trust property or assets in an adverse position or at risk of loss or damage.

17 MONITORING AND AUDITING

The Chief Investigator will ensure there are adequate quality and number of monitoring activities conducted by the study team. This will include adherence to the protocol, procedures for consenting and ensure adequate data quality. The Chief Investigator will inform the sponsor should he/she have concerns which have arisen from monitoring activities, and/or if there are problems with oversight/monitoring procedures.

18 TRAINING

The Chief Investigator will review and provide assurances of the training and experience of all staff working on this study. Appropriate training records will be maintained in the study files.

Our project researchers are psychology graduates. They have a degree in psychology but without clinical psychology training. They have been recruited on evidence of listening skills, empathy and dementia experience and have experience in qualitative work and have attended training and are supervised in their work. This level of expertise will ensure breadth of skills. A short training programme will be delivered by our leads. Project researchers have all completed UCL training in safety and diversity and training in Good Clinical Practice and informed consent. They will also have teaching sessions dedicated to cultural sensitivity. Training will emphasise the need to operate from an inclusive values base and to respect diversity and the existing knowledge and experience of those they interview.

19 INTELLECTUAL PROPERTY

Not applicable

20 INDEMNITY ARRANGEMENTS

University College London holds insurance against claims from participants for harm caused by their participation in this clinical study. Participants may be able to claim compensation if they can prove that UCL has been negligent. However, if this clinical study is being carried out in a hospital, the hospital continues to have a duty of care to the participant of the clinical study. University College London does not accept liability for any breach in the hospital's duty of care, or any negligence on the part of hospital employees. This applies whether the hospital is an NHS Trust or otherwise.

21 ARCHIVING

UCL and each participating site recognise that there is an obligation to archive study-related documents at the end of the study (as such end is defined within this protocol). The Chief Investigator confirms that he/she will archive the study master file at the Division of Psychiatry, Maple House for the period stipulated in the protocol and in line with all relevant legal and statutory requirements. The Principal Investigator at each participating site agrees to archive his/her

respective site's study documents for the duration of the study and in line with all relevant legal and statutory requirements.

22 PUBLICATION AND DISSEMINATION POLICY

Authorship will be decided as per usual group processes. Results will be disseminated through scientific peer-reviewed articles, conference presentations, and lay outputs such as update emails to participating site. The study will also maintain a website hosted by UCL and a Twitter account.

23 REFERENCES

1. Draper BM, Poulos CJ, Cole A, Poulos RG, Ehrlich F. A comparison of caregivers for elderly stroke and dementia victims. Journal of the American Geriatrics Society. 1992;40(9):896-901.
2. Ory MG, Hoffman III RR, Yee JL, Tennstedt S, Schulz R. Prevalence and impact of caregiving: A detailed comparison between dementia and nondementia caregivers. The Gerontologist. 1999;39(2):177-86.
3. Mahoney R, Regan C, Katona C, Livingston G. Anxiety and depression in family caregivers of people with Alzheimer disease: the LASER-AD study. The American Journal of Geriatric Psychiatry. 2005;13(9):795-801.
4. Cooper C, Balamurali TB, Livingston G. A systematic review of the prevalence and covariates of anxiety in caregivers of people with dementia. International psychogeriatrics. 2007;19(2):175-95.
5. Kinosian BP, Stallard E, Lee JH, Woodbury MA, Zbrozek AS, Glick HA. Predicting 10-Year Care Requirements for Older People with Suspected Alzheimer's Disease. Journal of the American Geriatrics Society. 2000;48(6):631-8.
6. Prince M, Prina M, Guerchet M. Journey of Caring: an analysis of long-term care for Dementia: N/A Ed; London: Alzheimer's Disease International; 2013.
7. Livingston G, Barber J, Rapaport P, Knapp M, Griffin M, Romeo R, et al. START (STrAtegies for RelaTives) study: a pragmatic randomised controlled trial to determine the clinical effectiveness and cost-effectiveness of a manual-based coping strategy programme in promoting the mental health of carers of people with dementia. Health technology assessment. 2014;18(61):1-242.
8. Livingston G, Kelly L, Lewis-Holmes E, Baio G, Morris S, Patel N, et al. Non-pharmacological interventions for agitation in dementia: systematic review of randomised controlled trials. The British Journal of Psychiatry. 2014;205(6):436-42.
9. Livingston G, Barber J, Rapaport P, Knapp M, Griffin M, King D, et al. Long-term clinical and cost-effectiveness of psychological intervention for family carers of people with dementia: a single-blind, randomised, controlled trial. The lancet Psychiatry. 2014;1(7):539-48.
10. Lord K, Rapaport P, Cooper C, Livingston G. Disseminating START: training clinical psychologists and admiral nurses as trainers in a psychosocial intervention for carers of people with dementia's depressive and anxiety symptoms. BMJ open. 2017;7(8):e017759.
11. Mukadam N, Cooper C, Basit B, Livingston G. Why do ethnic elders present later to UK dementia services? A qualitative study. International psychogeriatrics. 2011;23(7):1070-7.
12. Mukadam N, Cooper C, Livingston G. A systematic review of ethnicity and pathways to care in dementia. International journal of geriatric psychiatry. 2011;26(1):12-20.
13. Mukadam N, Waugh A, Cooper C, Livingston G. What would encourage help-seeking for memory problems among UK-based South Asians? A qualitative study. BMJ open. 2015;5(9):e007990.
14. Tuerk R, Sauer J. Dementia in a Black and minority ethnic population: characteristics of presentation to an inner London memory service. BJPsych Bulletin. 2015;39(4):162-6.

15. Cooper C, Tandy AR, Balamurali TB, Livingston G. A systematic review and meta-analysis of ethnic differences in use of dementia treatment, care, and research. The American Journal of Geriatric Psychiatry. 2010;18(3):193-203.
16. Berwald S, Roche M, Adelman S, Mukadam N, Livingston G. Black African and Caribbean British communities' perceptions of memory problems: "we don't do dementia.". PLoS One. 2016;11(4):e0151878.
17. Werner P, Mittelman MS, Goldstein D, Heinik J. Family stigma and caregiver burden in Alzheimer's disease. The Gerontologist. 2012;52(1):89-97.
18. Werner P, Goldstein D, Karpas DS, Chan L, Lai C. Help-seeking for dementia: a systematic review of the literature. Alzheimer Dis Assoc Disord. 2014;28(4):299-310.
19. Cheston R, Baghirathan S, Hui R, Shears P, Chacun A, Currie K. The dementia experiences of people from Caribbean, Chinese and South Asian communities in Bristol. Alzheimer's Society, 2017.
20. Parveen S, Blakey H, Oyebode J. Information Programme for South Asian families (IPSAF): An external evaluation. Bradford: 2015.
21. Health Do. Prime Minister's Challenge on Dementia 2020. Department of Health London, UK; 2015.
22. Damschroder LJ, Aron DC, Keith RE, Kirsh SR, Alexander JA, Lowery JC. Fostering implementation of health services research findings into practice: a consolidated framework for advancing implementation science. Implementation Science. 2009;4:50.
23. Kirk MA, Kelley C, Yankey N, Birken SA, Abadie B, Damschroder L. A systematic review of the use of the Consolidated Framework for Implementation Research. Implementation science : IS. 2016;11:72.
24. Graham ID, Logan J, Harrison MB, Straus SE, Tetroe J, Caswell W, et al. Lost in knowledge translation: time for a map? The Journal of continuing education in the health professions. 2006;26(1):13-24.
25. Moore GF, Audrey S, Barker M, Bond L, Bonell C, Hardeman W, et al. Process evaluation of complex interventions: Medical Research Council guidance. Bmj. 2015;350:h1258.
26. Snaith RP. The Hospital Anxiety And Depression Scale. Health and quality of life outcomes. 2003;1:29.
27. Bjelland I, Dahl AA, Haug TT, Neckelmann D. The validity of the Hospital Anxiety and Depression Scale: An updated literature review. Journal of psychosomatic research. 2002;52(2):69-77.
28. Pettit T, Livingston G, Manela M, Kitchen G, Katona C, Bowling A. Validation and normative data of health status measures in older people: the Islington study. International Journal of Geriatric Psychiatry

2001;16(11):1061-70.
29. Glasgow RE, Vogt TM, Boles SM. Evaluating the public health impact of health promotion interventions: the RE-AIM framework. American journal of public health. 1999;89(9):1322-7.
30. Gitlin LN, Marx K, Stanley IH, Hodgson N. Translating evidence-based dementia caregiving interventions into practice: state-of-the-science and next steps. The Gerontologist. 2015;55(2):210-26.
31. Patton M. Qualitative Research and Evaluation Methods. 3rd ed. Thousand Oaks: Sage Publications; 2002.
32. Miles MB, Huberman AM. Qualitative data analysis: an expanded sourcebook. Thousand Oaks: Sage Publications; 1994.
33. Beecham J, Knapp M. Costing psychiatric interventions. In: Thornicroft G, Brewin C, Wing J, editors. Measuring Mental Health Needs. Oxford: Oxford University Press; 1992.
34. Cooper C, Maxmin K, Selwood A, Blanchard M, Livingston G. The sensitivity and specificity of the Modified Conflict Tactics Scale for detecting clinically significant elder abuse. International psychogeriatrics. 2009;21(4):774-8.

35. Beach SR, Schulz R, Williamson GM, Miller LS, Weiner MF, Lance CE. Risk factors for potentially harmful informal caregiver behavior. Journal of the American Geriatrics Society. 2005;53(2):255-61.

24 APPENDICES

Please see files attached
