# Supplementary material for: Implementation of START (STrAtegies for RelaTives) for dementia carers in the third sector: Widening access to evidence-based interventions
Source: PLoS One. 2021 Jun 2;16(6):e0250410. doi: 10.1371/journal.pone.0250410 (PMC8171938; doi:10.1371/journal.pone.0250410)
Supplement: S1 Fig — (DOC) [file pone.0250410.s004.doc]

**
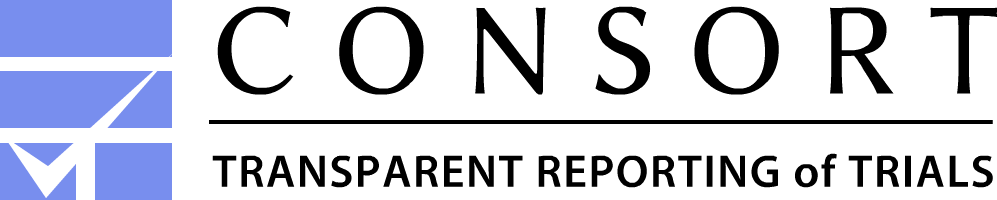
**

**CONSORT 2010 Flow Diagram🞴**

**Allocation**

**Analysis**

**Follow-Up**

**Enrollment**

Assessed for eligibility (n= 9 )

Excluded (n=0)

♦  Not meeting inclusion criteria (n=0)

♦  Declined to participate (n=0)

♦  Other reasons (n=0)

Analysed (n= 9)
♦ Excluded from analysis (give reasons) (n=0)

Lost to follow-up (give reasons) (n=0)

Discontinued intervention (give reasons) (n=0)

Allocated to intervention (n=9)

♦ Received allocated intervention (n=9)

♦ Did not receive allocated intervention (give reasons) (n=0)

Lost to follow-up (give reasons) (n=N/A)

Discontinued intervention (give reasons) (n= N/A )

Allocated to intervention (n=N/A)

♦ Received allocated intervention (n=N/A)

♦ Did not receive allocated intervention (give reasons) (n=N/A)

Analysed (n=N/A )
♦ Excluded from analysis (give reasons) (n= N/A )

Randomized (n=N/A)

🞴non-randomised design
